# Editorial: Problems, strategies, and developments for high-density long-term chronic intracortical neural interfaces and their application

**DOI:** 10.3389/fnins.2024.1373451

**Published:** 2024-02-13

**Authors:** João Filipe Ribeiro, Kenneth L. Shepard, Patrick Ruther

**Affiliations:** ^1^Microtechnology for Neuroelectronics Lab, Istituto Italiano di Tecnologia, Neuroscience and Brain Technologies, Genova, Italy; ^2^Departments of Electrical Engineering and Biomedical Engineering, Columbia University, New York, NY, United States; ^3^Department of Microsystems Engineering (IMTEK), University of Freiburg, Freiburg, Germany; ^4^BrainLinks-BrainTools Center, University of Freiburg, Freiburg, Germany

**Keywords:** bending stiffness, bio-interfaces, CMOS-based neural probes, electrode density, electrophysiological recording, implantation procedure, materials, electrical stimulation

The development of technical tools that can be applied in neuroscientific studies require highly interdisciplinary research, from materials science and microdevice development to animal experimentation and data analysis (Chen and Fang, 2023). Recently, a milestone was reached with high-density CMOS-based neural probes being used in a clinical trial (Paulk et al., 2022). Next generation brain computer interfaces (BCIs) require several technical developments at different levels but the need for long-term stability is always a key feature (Guo et al., 2022). This Frontiers Research Topic intends to provide a broad overview of the key aspects related to neuroengineering research and its latest's achievements. The four publications selected here span from: (i) 2D materials, particularly graphene, applied to neuroscience studies (Ye et al.), (ii) 3D orientation selective stimulation (OSS) applied to deep brain stimulation (DBS) studies (Gureviciene et al.), (iii) opto-electrodes for optogenetics stimulation studies under chronic conditions (Zhang et al.), and (iv) chronic CMOS-based neural interfaces, as well as their physiological constrains, probe-tissue interactions and perspectives for chronic large-scale, high resolution BCIs (Perna et al.).

The discovery of 2D nanomaterials and their distinctive properties is driving scientific advancements across various research fields. Graphene, among the large variety of these nanomaterials, stands out as the most extensively researched, especially in the realm of biomedical applications. Ye et al., provide an overview of the main graphene properties applied to biomedical applications and in particularly to neuroscience, such as mechanical, electrical, thermal, biocompatibility, and toxic proprieties. The biocompatibility and toxicity of graphene and how this depends on its functionalization are key aspects discussed by the authors. Although graphene-based materials can be applied from cell-cultures to *in-vivo* experiments, covering the central and peripheral nervous system, most of the currently available studies are in pre-clinical setups. Beyond its recording applications, graphene demonstrates the potential to foster neurite regrowth and repair damaged nerves, particularly in electrical stimulation setups designed to modulate neural activity.

Undoubtedly, stimulation capabilities to disturb neural circuits play a crucial role in intervening and aiding patients with neural-related disorders. While DBS is a common therapeutic intervention, its limitation lies in the lack of selectivity to precisely target brain circuits and avoid unintended effects in neighboring regions. In a groundbreaking contribution, Gureviciene et al. introduced the concept of 3D OSS enabling to direct the electric field at any angle. Employing a rat model for treatment-resistant depression, the study demonstrated significant improvements. The recorded local electrical responses in the amygdala correlated consistently with variations in stimulation field orientation, a validation accomplished through viral vectors and tractography using diffusion magnetic resonance imaging (MRI). The 3D OSS approach facilitates individualized DBS optimization through a single tetrahedral stimulation probe implantation for each patient. While the improved spatial stimulation selectivity achieved by the authors does not alter the non-selective activation of cells, it holds promise in avoiding stimulation of pathways with undesirable, often motor-related, effects and adapting the stimulation parameters to individual patients avoiding the common DBS approach of “*one fits all*.”

An alternative route to achieve stimulation selectivity involves the transition from electrical to optical methods. Optogenetics allows to use light as a stimulation trigger, achieved by pre-injecting opsins into target cells. While optogenetics offers advantages in selectivity, it also poses challenges such as the necessity of opsin injecting and the need for advancements of neuro interfaces to integrate light delivery capabilities. Zhang et al. introduced a mass-producible opto-electrode tested it in a freely moving mouse model. They developed a method to precisely laser cut microwires and assemble them with optical fibers (for light delivery) in a compact and lightweight manner. The resulting opto-electrode interface enables synchronous recording and stimulation across multiple brain regions, holding promise for advancing research on neural circuits and networks. Although the authors successfully recorded neural activity chronically for 5 weeks, they caution that the implant's rigidity may lead to persistent tissue damage and immunoreactive glial responses in the brain.

The long-term stability of implants is a paramount concern and a pivotal factor in implants failure. Perna et al. provide an extensive overview of crucial aspects associated with the stability and design of next-generation implants. First, the authors described and reviewed physiological constrains imposed by the brain, dividing them in three main considerations: the mechanical properties of the brain, brain micromotion and foreign body reactions (FBRs). Subsequently, they elaborate interactions between probe and brain tissue considering factors as the probe's Young's modulus, bending stiffness, and geometrical dimensions. The physicochemical properties of the probes and their implantation procedure were also discussed. Finally, active CMOS-based neural probes are placed in relation to traditional passive devices, highlighting the former's advantages in electrode density and spatial resolution. The authors conclude by presenting a forward-looking perspective on how this innovative technology is reshaping the landscape of intracortical electrophysiological recordings, envisioning enhancements chronic large-scale high-resolution BCIs.

## Author contributions

JR: Writing—original draft, Writing—review & editing. KS: Writing—review & editing. PR: Writing—original draft, Writing—review & editing.
